# Trichobezoar presenting with the 'comma sign' in Rapunzel Syndrome: a case report and literature review

**DOI:** 10.1186/1757-1626-1-286

**Published:** 2008-10-30

**Authors:** S Dindyal, NJ Bhuva, S Dindyal, MJ Ramdass, V Narayansingh

**Affiliations:** 1 Royal Cornwall Hospital NHS Trust, South West England, UK; 2 Imperial College Healthcare NHS Trust, London, UK; 3 Plymouth Hospital NHS Trust, South West England, UK; 4 South East Thames NHS Trust, London, UK; 5 General Hospital, Port-of-Spain, Trinidad, West Indies

## Abstract

**Introduction:**

Rapunzel Syndrome is an uncommon presentation of trichobezoar, involving strands of swallowed hair extending as a tail through the small intestine, beyond the stomach. This was first described in 1968 and until 2003 only thirteen cases have been reported.

**Case Presentation:**

A 55-year old man of East Indian descent presented to the surgical team via the emergency department in Trinidad, West Indies, with an acute abdomen and small bowel obstruction. He had a recent psychiatric history and patchy alopecia as well as a family history of schizophrenia. A supine abdominal radiograph revealed a small bowel obstruction as well as an unusual air shadow in the left hypochondrium in the region of the duodeno-jejunal flexure associated with the stomach bubble, which resembled a *'comma'*. At laparotomy, the stomach and third part of the duodenum were distended and contained a large mass of dark, foul-smelling hair that occupied the stomach, crossed the pylorus and extended into the small bowel. A retroperitoneal perforation of the third part of duodenum was found and repaired. The large trichobezoar was removed via a gastro-enterotomy and the patient made a slow, but complete recovery.

**Conclusion:**

We wish to report another case of Rapunzel Syndrome and describe an unusual radiologic sign associated with a retroperitoneal perforation of the third part of duodenum – *the comma sign*. To the best of our knowledge this is the first reported case of a spontaneous retroperitoneal perforation of the third part of duodenum associated with Rapunzel Syndrome.

## Introduction

Bezoars are collections of indigestible organic or inorganic foreign material in the gastrointestinal tract, which have usually accumulated in the stomach. Classification depends on composition and four types of bezoar have been described; phytobezoars, composed of vegetable matter, lactobezoars, which occur secondary to infant formula and consist of milk curd, pharmacobezoars or medication bezoars and trichobezoars, which are conglomerations of hair or hair like fibres. Rapunzel Syndrome is an uncommon presentation of trichobezoar, involving strands of swallowed hair extending as a tail through the small intestine, beyond the stomach (Rapunzel syndrome after 'Rapunzel' – the maiden in the Grimm brothers' fairy tale whose long hair flowed out of her prison tower allowing her prince to rescue her). *Vaughan et al *first described it in 1968 with only 13 cases formally reported in the literature to date [1-3,6-15]. We wish to report another case of Rapunzel Syndrome and describe an unusual radiological feature – *the comma sign*.

## Case presentation

A 55-year old man of East Indian descent presented as an emergency to the surgical team via the emergency department in Trinidad, West Indies, with diffuse abdominal pain, fever, nausea and constipation. The abdominal pain was generalised and colicky in nature, and the patient was most tender in the peri-umbilical region. He had a recent psychiatric history and patchy alopecia as well as a family history of schizophrenia.

**Figure 1:**
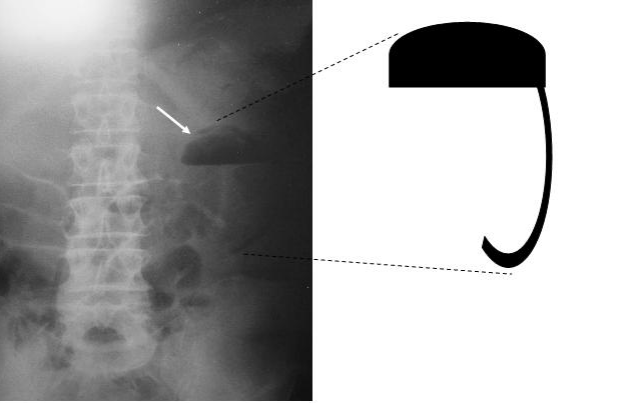
Abdominal radiograph of the 'comma sign' in our patient.

On examination the patient was pyrexial and tachycardic with a hard periumbilical abdominal mass as well as generalised tenderness and guarding. Bilateral inguinal hernias were found to be easily reducible. A supine abdominal radiograph revealed a small bowel obstruction associated with an unusual air shadow in the left hypochondrium in the region of the stomach and duodeno-jejunal flexure, which resembled a *'comma' *[Figure 1]. Unfortunately a Computed Tomography (CT) scanner was not available at the time and transferring the patient to a larger hospital to obtain an abdominal CT scan would have jeopardised his survival in the context of an acute abdomen.

At laparotomy the stomach, third & fourth parts of the duodenum were distended and contained a large mass of dark, foul smelling hair that had crossed the pylorus and extended into the small bowel. There was also a retroperitoneal perforation of the third part of the duodenum with air in this space. The large trichobezoar was removed via a gastro-enterotomy. Recovery was slow but complete and the patient was eventually discharged.

## Discussion

Trichobezoars, although rare, are most common in children and young women. DeBakey and Ochsner looked at 172 cases of trichobezoar and found that almost 90% occurred in teenage females [4]. There is an association with mental retardation and psychiatric conditions; almost half of patients present with trichophagia [5]. Trichobezoar formation begins with small pieces of hair, which gather in the stomach as they are ingested but do not progress any further. As the mass builds up the stomach is unable to dislodge it. Sometimes, the aggregate of hair fragments and the small pieces that result do pass through into the intestine, thus leading to sequelae such as ulceration, partial or total obstruction, intestinal perforation and peritonitis. Patients are often asymptomatic, but may present with nausea, vomiting, anorexia, weight loss, vague abdominal pain or constipation.

Rapunzel Syndrome occurs when the trichobezoar has extended into the duodenum and small intestine and manifests itself with nausea, vomiting, anorexia and weight loss [6].

Rapunzel Syndrome was first reported in the West Indies by *Duncan et al *in 1994 [7]. The diagnosis is based on a combination of good history taking as well as physical findings to look for a family history of psychiatric disorders, previous bezoars, a palpable mass, patchy hair loss and halitosis.

**Figure 2:**
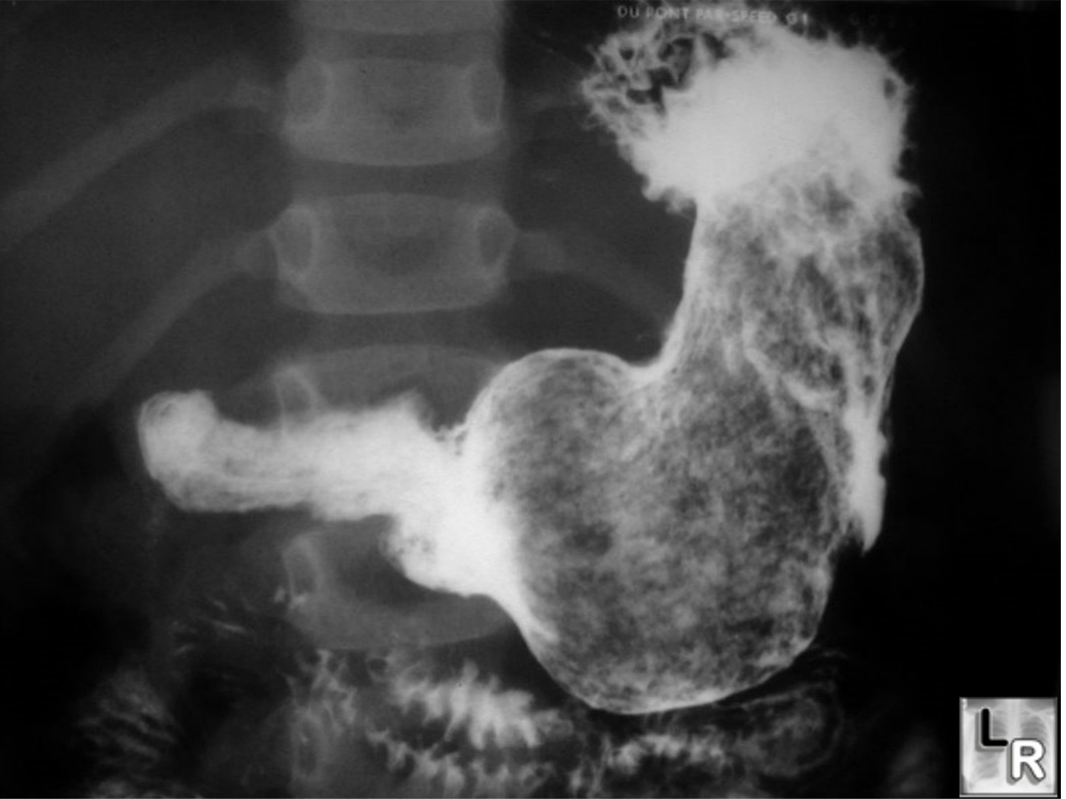
**Barium meal showing filling defect due to trichobezoar. **.

The use of ultrasound (US), CT scanning and endoscopy have vital roles, and the trichobezoar may appear as a gastric obstruction and palpable mass in the epigastrium. The upper part of a large bezoar may be visible as a mass with a convex upper border projecting into the gastric air bubble. An erect abdominal radiograph and a supine radiograph may also show a prominent gastric outline with an intragastric mottled mass, outlined by gas in the distended stomach, mimicking a food-filled stomach. With a small amount of barium the hair-ball gets coated and becomes visible. Filling the stomach with barium demonstrates a mobile intraluminal filling defect of variable size, which may show extension into the duodenum [Figure 2].

Abdominal Ultrasound shows a dense, echogenic rim with sharp, clear posterior acoustic shadowing in the epigastric region. This characteristic appearance persists irrespective of the angulation of the transducer or alteration of position of the patient. This can be attributed to multiple tiny interfaces between the smooth, compressed, compact mass and the entrapped air and food debris. This specific US appearance excludes the clinical possibility of a pancreatic pseudocyst, splenic or renal mass, non-calcified gastric tumour, gastric duplication cyst and gastric outlet obstruction. However a heavily calcified mass such as teratoma, neuroblastoma or impacted mass of faeces may produce a similar US image.

**Figure 3:**
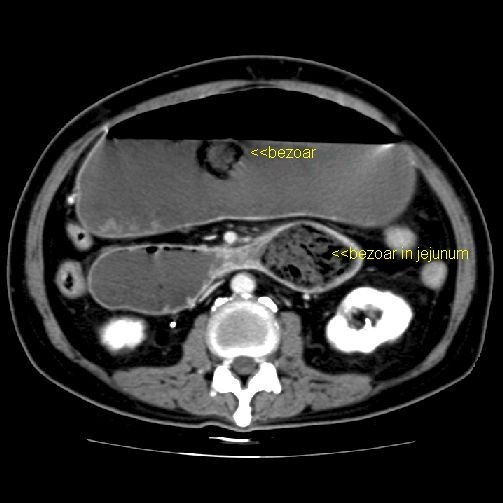
**CT image of trichobezoar.** [K M. Vitellas, K Vaswani and W F. Bennett: Small-Bowel Bezoar *AJR *2000; 175:876–878].

Plain abdominal CT usually shows a mobile intragastric mass consisting of "compressed concentric rings", with a mixed density pattern due to the presence of entrapped air and food debris [Figure 3]. Often small collections of barium from a previous upper gastrointestinal barium study may be interspersed within the mass.

Perforation resulting from such obstruction appears traditionally as free air under the diaphragm on an erect chest radiograph, with a mottled gas pattern over the stomach resembling pneumatosis intestinalis (gas in the wall of the intestine). CT appearances show pneumoperitoneum and curvilinear, serpiginous, bubbly collections of gas that occur circumferentially or in peripheral or dependent portions of the bowel wall with long segments bowel usually affected.

The diagnosis can also be established by endoscopy, where trichobezoars are often found to have a hard, concrete like appearance and enzymatic oxidation of hair gives it a darkened colour. Treatment may include endoscopic removal, chemical dissolution and mechanical fragmentation, for small bezoars. However, surgery is sometimes necessary for large bezoars with extension into the bowel as seen in this case. The bezoar plus its tail can be removed via gastrotomy and sometimes multiple enterotomies to reduce risk of gut perforation, as long bezoar tails are often extremely adherent to the side of the gut wall [8]. In this case a gastrotomy with single enterotomy was sufficient to remove the trichobezoar. Laparascopic techniques are also becoming fashionable and large bezoars can be milked into the caecum before removal in this manner.

*Kumar et al *first reported recurrent trichobezoar in 1996, where it was seen to occur because the underlying emotional stress trigger was not corrected [11]. Recurrence of Rapunzel Syndrome has not been discussed at any great length in the literature and there is only one other report by *Memon et al *in 2003, the cause being non-compliance of the patient regarding her psychiatric medication [13]. Indeed, treatment and psychological support of the mental as well of physical disorder is important. Trichotillomania is a facet of obsessive-compulsive disorder and can be treated with tricyclic antidepressants.

## Conclusion

We report this further case of Rapunzel Syndrome associated with a small bowel obstruction. Additionally, we learnt in restrospect that the radiographic appearance of the *'comma' *in the left upper quadrant was actually the stomach bubble (with the bezoar in it) and air in the retroperitoneal space surrounding the perforation of the third part of duodenum and duodeno-jejunal flexure thereby simulating a *'comma'*.

Of all the cases of Rapunzel Syndrome described in literature with free perforations, these cases presented with acute abdomens and free air under the diaphragm. We would therefore like to describe the *'comma sign' *associated with Rapunzel Syndrome for the first time. We also acknowledge the fact that the *'comma sign' *may apply to any patient with a retroperitoneal perforation of the third part of the duodenum. However, this by itself is quite a rare occurrence and when identified on a plain supine abdominal radiograph should alert the clinician to rare pathology such as tumour, foreign body or trichobezoar.

We recommend closer attention be paid to the appearance of this sign on a plain supine abdominal radiograph in order to facilitate greater recognition of retroperitoneal perforations in this region and to correlate the findings with the history in order to diagnose bezoars of the Rapunzel Syndrome type pre-operatively.

We hope this case report reminds and helps our emergency medicine and surgical colleagues with the diagnosis and treatment of this rare and unusual condition in the future.

## Consent

Written informed consent was obtained from the patient for publication of this case report and accompanying photographic and radiographic images.

## Competing interests

The authors declare that they have no competing interests.

## Authors' contributions

SD reported the case, provided the imaging and helped to draft the manuscript. NB drafted the manuscript, researched the factual content and literature and revised it critically for important intellectual substance. PS oversaw the project and helped draft the manuscript. VN oversaw the project and helped draft the manuscript. All authors read and approved the final manuscript.
